# From Physicochemical Classification to Multidimensional Insights: A Comprehensive Review of Uremic Toxin Research

**DOI:** 10.3390/toxins17060295

**Published:** 2025-06-10

**Authors:** Mario Cozzolino, Lorenza Magagnoli, Paola Ciceri

**Affiliations:** Department of Health Sciences, University of Milan, 20122 Milan, Italy; lorenza.magagnoli@unimi.it (L.M.); paola.ciceri@unimi.it (P.C.)

**Keywords:** uremic toxins, chronic kidney disease, hemodialysis, hemoadsorption, toxicity mechanisms, precision medicine

## Abstract

Chronic kidney disease (CKD) is a global health burden, with uremic toxins (UTs) playing a central role in its pathophysiology. In this review, we systematically examined the evolution of UT classification from the 2003 European Uremic Toxin Work Group (EUTox) system based on molecular weight and protein-binding properties to the 2023 multidimensional framework integrating clinical outcomes, clearance technologies, and artificial intelligence. We highlighted the toxicity mechanisms of UTs across the cardiovascular, immune, and nervous systems and evaluated traditional (e.g., low-/high-flux hemodialysis) and advanced (e.g., high-cutoff dialysis and hemoadsorption) clearance strategies. Despite progress, challenges persist in toxin detection, clearance efficiency, and personalized therapy. Future directions include multi-omics-based biomarker discovery, optimized dialysis membranes, advanced adsorption technology, and AI-driven treatment personalization. This synthesis aims to bridge translational gaps and guide precision medicine in nephrology.

## 1. Introduction

### 1.1. Background of Uremic Toxins

Chronic kidney disease (CKD) is a global health concern, with its prevalence increasing significantly worldwide, affecting over 10% of the global population, with uremic toxin accumulation driving morbidity and mortality [1]. The accumulation of uremic toxins is a key challenge in CKD management. These toxins, derived from normal metabolism and gut microbiota-related processes, contribute to a range of pathophysiological changes and clinical symptoms in patients. Recent advances in toxin identification and isolation technologies, such as proteomics and metabolomics, have provided new insights into the nature and impact of these toxins [2,3]. Additionally, the role of the gut microbiome in toxin production is an emerging area of research with significant potential for understanding and managing CKD.

The study of uremic toxins has a long-standing history. The concept of uremia was first proposed in the 19th century. With the development of medical technology, our understanding of uremic toxins has gradually deepened. Uremic toxins were initially considered to mainly be simple waste products, and the focus was on removing them through dialysis. However, with in-depth research, it has been found that uremic toxins are a complex mixture of substances with diverse chemical structures and properties, and they play crucial roles in the progression of CKD and the occurrence of related complications.

Uremic toxins have a significant impact on patient health. They can affect multiple systems in the body, such as the cardiovascular [2,3], nervous, immune [3], and endocrine systems. For example, some uremic toxins can cause vascular endothelial damage, accelerate atherosclerosis, and increase the risk of cardiovascular events, which are one of the main causes of high mortality in uremic patients (a study in 2021 showed that cardiovascular mortality accounts for ≈40% to 50% of all deaths in patients with CKD stages 4 and 5 [4]). In the nervous system, uremic toxins can lead to uremic encephalopathy, which manifests as cognitive impairment, seizures, and other symptoms, seriously affecting the quality of life of patients. In addition, uremic toxins can also disrupt the immune balance, making patients more susceptible to infections, and they can interfere with the normal function of the endocrine system, leading to disorders of calcium–phosphorus metabolism and anemia.

With the continuous increase in the global incidence of CKD, the number of patients with uremia is also increasing [1]. According to the latest data from the Global Burden of Disease Study, the prevalence of CKD has reached high levels, and a large number of patients eventually progress to end-stage renal disease (ESRD), which requires renal replacement therapy. Therefore, understanding the classification and clearance mechanisms of uremic toxins is of great significance for improving the treatment effect and quality of life of uremic patients, and it has become a hot topic in the field of nephrology research.

### 1.2. Research Significance and Purpose

The study of uremic toxins is significant for several reasons. Firstly, a deep understanding of uremic toxins can help medical professionals better understand the pathophysiological mechanisms of CKD [4]. Different types of uremic toxins have unique effects on the body, and by clarifying these effects, it will be possible to uncover the underlying causes of various complications in uremic patients, such as cardiovascular disease, neuropathy, and immune dysfunction. This understanding can provide a theoretical basis for the development of more targeted treatment strategies.

Secondly, improving the classification and assessment of uremic toxins is crucial for optimizing treatment plans. In clinical practice, the accurate classification of toxins can help doctors select the most appropriate treatment methods, such as hemodialysis (HD) or hemoadsorption (HA), according to the characteristics of different toxins. For example, for protein-bound uremic toxins (PBUTs) that are difficult to clear using traditional dialysis, specific hemoadsorption techniques may be required. Moreover, a more accurate assessment of toxin toxicity can help doctors better evaluate the treatment effect and adjust the treatment plan in a timely manner.

Finally, the study of uremic toxins also has important implications for the development of new drugs and treatment technologies. By understanding the structure and function of uremic toxins, researchers can design drugs that specifically target these toxins, or they can develop new blood purification technologies with higher efficiency and selectivity. This can not only improve the treatment effect of uremic patients but also reduce the treatment burden and improve their quality of life.

The purpose of this review is to comprehensively examine the research progress in the classification, toxicity assessment, and clearance technologies of uremic toxins. By analyzing the existing literature, this review aims to summarize the current understanding of uremic toxins, including the classification methods proposed in different periods, the assessment of toxin toxicity, and the research status of various clearance technologies such as HD and HA. This will provide a reference for clinical treatment and future research, helping medical workers to better understand and handle uremic toxins, as well as promoting the development of the field of nephrology.

## 2. Classification of Uremic Toxins

### 2.1. Traditional Classification Based on Physicochemical Properties [5]

In 2003, the European Uremic Toxin Work Group (EUTox) proposed a classification system for uremic toxins, and it had a significant impact on the field of nephrology research. The classification principles of the 2003 EUTox system are mainly based on the molecular mass and protein-binding characteristics of toxins.

#### 2.1.1. Small-Molecule Toxins

Small-molecule toxins, which have a molecular weight of <500 Da, are an important category of uremic toxins. There are about 45 types of such toxins. They are mainly present in plasma in a free form, and most of them are water-soluble. This water solubility property enables them to be relatively easily cleared using conventional dialysis methods. For example, creatinine, a metabolite of muscle degradation with a molecular weight of 113 Da, is often used as a marker of renal function. In uremic patients, creatinine accumulates in the body due to impaired kidney function. Although it can be cleared to a certain extent using hemodialysis, its high—level presence still indicates the decline of renal function. Urea, with a molecular weight of 60 Da, is a by-product of protein metabolism. In healthy individuals, the kidneys can effectively excrete urea; however, in uremic patients, urea accumulates, reaching high concentrations in the blood. While urea and creatinine are not themselves uremic toxins, they serve as surrogates for the myriad low molecular weight water-soluble toxins that accumulate in uremia. Uric acid, which has a molecular weight of 168 Da and belongs to the purine category, is also a small-molecule toxin. In uremic patients, the reduced excretion of uric acid by the kidneys can lead to hyperuricemia, which may further cause gout and other complications.

Some small-molecule toxins, such as asymmetric dimethylarginine (ADMA), have strong biological toxicity [6]. ADMA, which has a molecular weight of 202 Da and belongs to the guanidine category, can inhibit nitric oxide (NO) synthesis, leading to vascular endothelial cell dysfunction and increased vascular resistance, ultimately contributing to the development of hypertension and cardiovascular diseases. High levels of ADMA in uremic patients are associated with a higher risk of cardiovascular events. These small-molecule toxins, despite their small molecular size, play important roles in the pathophysiological processes of uremia, and their effective clearance is crucial for the treatment of uremic patients.

#### 2.1.2. Middle-Molecule Toxins

Middle-molecule toxins, which have a molecular weight of ≥500 Da, are mainly derived from endogenous metabolism [7]. There are approximately 22 types of these toxins, and they mostly consist of peptides and cytokines. For example, β_2_-microglobulin (B2M) is a well-known middle-molecule toxin with a molecular weight of 11,818 Da. It is a component of major histocompatibility complex class I and is produced by most nucleated cells. In uremic patients, due to the inability of the kidneys to effectively clear B2M, it accumulates in the body, leading to dialysis-related amyloidosis, which can cause joint pain, carpal tunnel syndrome, and other symptoms.

Parathyroid hormone (PTH) is another important middle-molecule toxin, with a molecular weight of 9225 Da. PTH is secreted by the parathyroid gland, and it plays a crucial role in regulating calcium–phosphorus metabolism. In uremic patients, the disorder of calcium–phosphorus metabolism leads to secondary hyperparathyroidism, with an increased secretion of PTH. High levels of PTH can cause bone resorption, osteoporosis, and soft tissue calcification, seriously affecting bone and mineral metabolism in patients.

Interleukin-6 (IL-6), a cytokine with a molecular weight of 24,500 Da, is also a middle-molecule toxin. In uremic patients, the inflammatory state often leads to increased levels of IL-6. Elevated IL-6 can promote chronic inflammation, accelerate the progression of atherosclerosis, and increase the risk of cardiovascular diseases.

Low-flux hemodialysis (LFHD) has a limited ability to clear middle-molecule toxins. This is because the pore size of the dialysis membrane in LFHD is relatively small, restricting the passage of larger-sized molecules. Compared to LFHD, high-flux hemodialysis (HFHD) has a certain improvement in the clearance of middle-molecule toxins. The larger pore size of the membrane in HFHD allows for a higher flux of solutes, enabling it to remove some middle-molecule toxins to a certain extent. However, for some larger middle-molecule toxins, the clearance efficiency of HFHD remains limited. Therefore, for the effective clearance of middle-molecule toxins, more advanced blood purification technologies, such as high-cutoff dialysis or adsorption-based methods (hemoadsorption), are often required.

#### 2.1.3. Protein-Bound Toxins

Protein-bound toxins are formed when toxins bind to proteins, resulting in the formation of ultra-large molecules, of which there are around 25 types [8]. These toxins are primarily derived from gut microbial metabolism. For example, indoxyl sulfate (IS) is a typical protein-bound toxin. It is produced by the metabolism of tryptophan by gut microbiota. Tryptophan is converted into indole by gut bacteria, and then indole is absorbed into the bloodstream and oxidized to indoxyl sulfate in the liver. IS has a molecular weight of 251 Da and a high protein-binding rate (more than 80%). Once bound to proteins, its water solubility decreases, and it becomes difficult to clear using traditional dialysis methods. Another protein-bound toxin, p-cresol sulfate (PCS), is produced by the metabolism of tyrosine by gut microbiota. PCS has a molecular weight of 108 Da, and it also has a high protein-binding rate. However, the free (unbound) component can be removed by HD, albeit to a limited extent due to its relatively low proportion.

The high protein-binding rate of these toxins significantly affects their dialysis clearance. As traditional dialysis mainly relies on diffusion and convection, the bound form of these toxins cannot easily pass through the dialysis membrane. As a result, the dialysis clearance rate of protein-bound toxins is much lower than that of non-bound toxins. For example, in a study by Vanholder et al. (2003) [5], it was found that the clearance of protein-bound toxins, such as IS and PCS, is extremely limited in conventional hemodialysis.

Protein-bound toxins are closely associated with chronic kidney disease (CKD), cardiovascular disease (CVD), and metabolic disorders. IS has been shown to cause endothelial cell damage, promote oxidative stress, and enhance the expression of inflammatory factors, all of which contribute to the development of atherosclerosis and cardiovascular diseases. PCS can also disrupt cell function and metabolism, leading to tissue damage and the progression of various diseases. In addition, these toxins can interfere with the normal function of the endocrine system, glucose metabolism, and lipid metabolism, further aggravating the metabolic disorders in uremic patients. Therefore, the effective removal of protein-bound toxins is a major challenge in uremia treatment, and it requires the development of specific clearance strategies, such as hemoadsorption, which utilizes porous resin or polymer-based adsorbents to selectively bind and immobilize these toxins via molecular sieves, hydrophobic interactions, and electrostatic forces as blood circulates through the hemoadsorption column, effectively separating them from the patient’s bloodstream.

The three main categories described above are presented in Table 1.

### 2.2. Evolution of Classification Systems

#### 2.2.1. The 2003 EUTox Classification [5]

In the 2003 EUTox classification, uremic toxins are divided into three main categories, which are described above.

However, the 2003 EUTox classification has some limitations. Firstly, the classification is mainly based on physicochemical properties, and the biological activity and clinical significance of toxins are not fully considered. For example, some toxins with relatively low concentrations but high biological activity may not be given sufficient attention. Secondly, the classification does not take into account the complex interactions between toxins and the body’s various systems. In fact, uremic toxins often interact with multiple physiological systems, and their effects on the body are not simply determined by their molecular mass and protein-binding characteristics. The interactions between different toxins themselves are complex and can significantly influence their overall toxic effects. For instance, certain toxins may synergistically enhance the toxicity of others. One well—documented example is the interaction between indoxyl sulfate (IS) and p-cresol sulfate (PCS). Both are protein-bound uremic toxins that can cause endothelial dysfunction and promote inflammation. Research has shown that when present together, their combined effects on vascular endothelial cells can be more pronounced than when either toxin is present alone. This synergistic interaction may exacerbate the development of cardiovascular diseases in uremic patients. Additionally, the presence of one toxin may affect the metabolism or clearance of another. Thirdly, with the development of new blood purification technologies, the classification, which is based on molecular mass and protein-binding alone, cannot fully meet the needs of clinical practice. For instance, new dialysis membranes and clearance techniques might have different effects on the removal of toxins, which are not well reflected in the 2003 EUTox classification.

#### 2.2.2. The 2021 Consensus Classification [9]

In 2021, a new consensus classification of uremic toxins was proposed, and it is an important improvement based on the 2003 EUTox classification [10]. This consensus classification takes into account multiple factors in addition to the physicochemical properties of toxins.

One of the significant improvements is the consideration of toxin clearance technologies and efficiency. It is recognized that different toxins have different clearance characteristics, and the choice of clearance technology should be based on the specific properties of the toxins. For example, for protein-bound toxins, traditional dialysis methods have limited clearance efficiency, so new technologies such as adsorption-based methods are needed. The 2021 consensus classification emphasizes the importance of understanding the relationship between toxin properties and clearance technologies to optimize the treatment of uremic patients.

Another important aspect is the consideration of the impact of toxins on target organs. Uremic toxins can affect multiple organ systems, such as the cardiovascular, nervous, immune, and endocrine systems. The 2021 consensus classification aimed to classify toxins according to their effects on different target organs. For example, some toxins that have a significant impact on the cardiovascular system, such as indoxyl sulfate and ADMA, are given special attention. By classifying toxins in this way, it became possible to better understand the pathophysiological mechanisms of uremic complications and develop more targeted treatment strategies.

Moreover, the 2021 consensus classification also considers clinical outcomes. It emphasizes that the classification of uremic toxins should be related to the actual clinical situation of the patient, such as their symptoms, prognosis, and quality of life. For example, toxins that are closely related to symptoms such as pruritus and restless leg syndrome are identified, and the classification aims to provide a basis for improving the treatment of these symptoms. This patient-centered approach is a major advancement in the classification of uremic toxins, as it focuses on the real-world impact of toxins on patients.

In summary, the 2021 consensus classification is more comprehensive and clinically relevant than the 2003 EUTox classification (Table 2 demonstrates the differences between them). It provides a more in-depth understanding of uremic toxins from multiple perspectives, including clearance technologies, target organ effects, and clinical outcomes (Table 3). This classification system is more conducive to guiding clinical practice and promoting the development of new treatment methods for uremic patients.

#### 2.2.3. The 2023 New Classification Dimensions [11]

**Figure 1:**
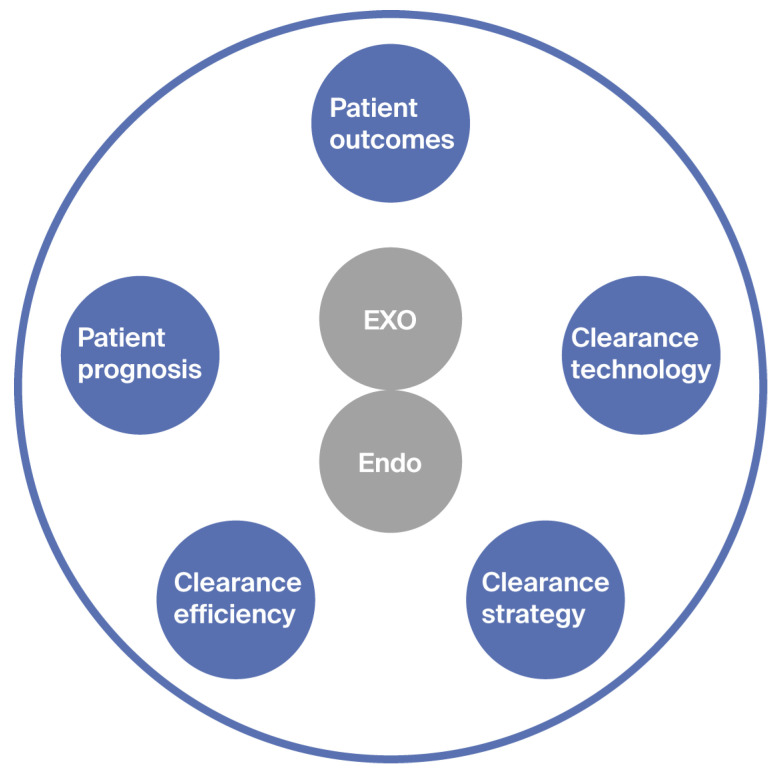
Multidimensional classification of uremic toxins based on molecular properties, clinical impact, and clearance technologies. This figure illustrates the integration of various factors in the classification of uremic toxins, highlighting the importance of considering molecular properties, clinical impact on different organ systems, and the efficiency of different clearance technologies.

In 2023, new classification dimensions for uremic toxins were proposed, further enriching and deepening the understanding of uremic toxins (Figure 1). One of the key focuses of this classification is patient outcomes. This new classification aims to assess the impact of known uremic toxins on patient-related outcomes, including quality of life (QoL) and quality-adjusted life-years. By evaluating the relationship between toxins and patient outcomes, it is possible to identify the most critical toxins that have a significant impact on patient well-being and survival. For example, toxins that are closely related to the occurrence of cardiovascular events, which are a major cause of mortality in uremic patients, are given special attention. Understanding the impact of these toxins on patient outcomes could help medical workers develop more effective treatment strategies to improve patient prognosis.

Another important aspect is the identification of novel uremic toxins that could impact patient outcomes. With the development of research technologies, an increasing number of new toxins are being discovered. The 2023 classification encourages the exploration of these novel toxins and their potential effects on patients. This could lead to a better understanding of the pathophysiological mechanisms of uremia and the discovery of new treatment targets. For example, some newly discovered toxins might be involved in the progression of chronic inflammation or the development of metabolic disorders in uremic patients. Identifying these toxins could provide new insights into the treatment of uremia.

The development, validation, and enhancement of dialyzer membranes with a tunable ability to preferentially remove different classes of uremic toxins are also emphasized in the 2023 classification. The different types of toxins have different molecular structures and properties, and traditional dialyzer membranes might not be able to effectively remove all of them. The new classification promotes the research and development of novel dialyzer membranes that could be customized to target specific classes of toxins. For example, membranes with specific pore sizes, surface properties, or chemical modifications could be designed to improve the clearance of protein-bound or medium-molecule toxins. This could significantly improve the efficiency of blood purification and the treatment effect on uremic patients.

In addition, the development, validation, and implementation of artificial intelligence and machine learning models to predict patient outcomes based on uremic toxin profiles and to match individual patients with appropriate dialysis strategies are also important aspects of the 2023 classification. By using artificial intelligence and machine learning models, it is possible to predict the outcomes of patients more accurately and select the most suitable dialysis strategies for individual patients. For example, these models could analyze the relationship between toxin levels and the occurrence of complications in a patient and recommend personalized dialysis schedules and treatment methods. This could improve the treatment precision and effectiveness for uremic patients, ultimately improving their quality of life and survival.

## 3. Toxicity Assessment of Uremic Toxins

### 3.1. Evaluation Indicators [5]

#### 3.1.1. C_U_/C_N_ Ratio

The C_U_/C_N_ ratio is an important indicator for evaluating the toxicity of uremic toxins [12]. C_U_ represents the mean/median uremic concentration of a toxin in uremic patients, while C_N_ represents the normal concentration of the toxin in healthy individuals. The principle behind using this ratio to assess toxicity is that a high C_U_/C_N_ ratio indicates a significant difference in the concentration of the toxin between uremic patients and healthy individuals. A high-ratio toxin has a much higher concentration in uremic patients. For example, as shown in a study by Vanholder et al. (2003), guanidinosuccinic acid has a CU/CN ratio of 216.67, and methylguanidine has a ratio of 106.00. Other toxins such as p-cresol sulfate (PCS) and trimethylamine N-oxide (TMAO) also have significant CU/CN ratios, indicating their potential contribution to toxicity [5]. These high-ratio toxins are often major contributors to toxicity.

Toxins with a high C_U_/C_N_ ratio can have toxic effects on multiple physiological systems. Protein-bound toxins, such as indoxyl sulfate (IS) and p-cresol sulfate (PCS), which often have high C_U_/C_N_ ratios, can cause damage to the cardiovascular system. IS, for instance, can promote oxidative stress, inflammation, and endothelial cell damage in the blood vessels, leading to the development of atherosclerosis and an increased risk of cardiovascular events. Some medium-molecule and water-soluble toxins with high C_U_/C_N_ ratios also play roles in disrupting physiological functions. For example, certain cytokines such as interleukin-6 (IL-6) with a relatively high C_U_/C_N_ ratio can exacerbate chronic inflammation, which not only affects the immune system but also has a negative impact on the cardiovascular and metabolic systems. High-C_U_/C_N_-ratio toxins are important factors in the pathophysiological processes of uremia, and their high-level presence in uremic patients highlights the need for effective clearance strategies.

#### 3.1.2. C_MAX_/C_U_ Ratio

The C_MAX_/C_U_ ratio is used to assess the uniformity of the distribution of a particular toxin. C_MAX_ represents the highest uremic concentration ever reported for a toxin in uremic patients. A high C_MAX_/C_U_ ratio indicates that the distribution of the toxin is uneven, and it may reach extremely high levels in specific patients.

For example, γ-guanidinobutyric acid has a C_MAX_/C_U_ ratio of 52.55, and 2-methoxyresorcinol has a ratio of 16.43. Such high-ratio toxins pose greater risks in certain patient populations. A high C_MAX_/C_U_ ratio may be influenced by factors such as genetics, protein binding, or gut microbiota. In terms of genetics, different genetic backgrounds may lead to differences in toxin metabolism and clearance, resulting in an uneven distribution. Protein-bound toxins, due to their strong binding to proteins, may have different release and metabolism rates among patients, leading to extreme accumulations in some individuals. Gut microbiota also play a role. Variations in the gut microbiota composition can affect toxin production and metabolism. For example, certain gut bacteria may produce more of a particular toxin in some patients, leading to a higher C_MAX_ in those individuals. Toxins with a high C_MAX_/C_U_ ratio need to be closely monitored, and personalized treatment strategies may be required to address the potential risks that they pose to specific patients.

### 3.2. Toxicity Manifestations and Mechanisms [6,13]

#### 3.2.1. Impact on the Cardiovascular System

Uremic toxins have a significant impact on the cardiovascular system, and this is one of the main causes of high mortality in uremic patients. Asymmetric dimethylarginine (ADMA) is a typical uremic toxin that can cause severe damage to the cardiovascular system. ADMA is a metabolite of arginine and is mainly excreted by the kidneys. In uremic patients, due to the decline of kidney function, ADMA accumulates in the body. ADMA can competitively inhibit the activity of nitric oxide synthase (NOS), which is an enzyme responsible for the synthesis of nitric oxide (NO) from L-arginine [14]. NO is a crucial molecule for maintaining vascular endothelial function. It can relax blood vessels, inhibit platelet aggregation, and reduce the adhesion of inflammatory cells to the endothelium. When ADMA inhibits NOS, NO production is reduced, leading to endothelial cell dysfunction. Endothelial cells lose their normal anti-inflammatory and anti-thrombotic properties, and the blood vessels become more prone to vasoconstriction, thrombosis, and inflammation. This can accelerate the development of atherosclerosis, increase blood pressure, and ultimately increase the risk of cardiovascular events such as myocardial infarction and stroke.

Indoxyl sulfate (IS) is another uremic toxin that has a major impact on the cardiovascular system. IS is produced by the metabolism of tryptophan by gut microbiota. It has a high protein-binding rate and is difficult to clear using traditional dialysis. IS can cause oxidative stress in vascular endothelial cells. It promotes the generation of reactive oxygen species (ROS), such as superoxide anion and hydrogen peroxide. These ROS can damage the cell membrane, DNA, and endothelial cell proteins, leading to cell apoptosis and dysfunction. IS can also activate the nuclear factor-κB (NF-κB) signaling pathway in endothelial cells. NF-κB is a transcription factor that can regulate the expression of various inflammatory cytokines, such as interleukin-6 (IL-6), tumor necrosis factor-α (TNF-α), and intercellular adhesion molecule-1 (ICAM-1). The activation of NF-κB by IS leads to the upregulation of these inflammatory cytokines, which can attract inflammatory cells to the blood vessels, promote inflammation, and accelerate the development of atherosclerosis. In addition, IS can induce the differentiation of vascular smooth muscle cells into osteoblast-like cells, leading to vascular calcification, which further increases the risk of cardiovascular disease.

#### 3.2.2. Influence on the Immune System

Uremic toxins can disrupt the normal function of the immune system, making uremic patients more susceptible to infections and causing immune function disorders. The accumulation of uremic toxins can lead to the activation of immune cells, such as monocytes and macrophages [15]. These activated immune cells produce excessive inflammatory cytokines, such as TNF-α, IL-1, and IL-6. The over-production of these cytokines can cause a systemic inflammatory response, which is called “uremic inflammation”. This chronic inflammatory state not only weakens the body’s immune defense ability but also promotes the progression of various complications, such as cardiovascular disease and anemia.

Some uremic toxins can directly inhibit the function of immune cells. For example, guanidinosuccinic acid, a small-molecule uremic toxin, can inhibit the proliferation and function of lymphocytes. Lymphocytes are important immune cells that play a crucial role in adaptive immunity. The inhibition of lymphocyte function by guanidinosuccinic acid can reduce the body’s ability to recognize and respond to pathogens, making patients more vulnerable to infections.

Uremic toxins can also affect the function of the complement system. The complement system is an important part of the innate immune system, which can be activated by pathogens or immune complexes to kill pathogens and promote inflammation. Uremic toxins can cause the abnormal activation of the complement system, leading to the generation of excessive complement components, such as C3a and C5a. These complement components can cause inflammation, tissue damage, and immune-mediated diseases. In addition, the abnormal activation of the complement system can also lead to the consumption of complement components, reducing the body’s immune defense ability.

#### 3.2.3. Effects on the Nervous System

Guanidine-type toxins, such as methylguanidine and guanidinosuccinic acid, are important uremic toxins that can cause damage to the nervous system. Methylguanidine can cross the blood–brain barrier and accumulate in the brain. It can interfere with the normal function of neurons by affecting neurotransmitter metabolism. For example, methylguanidine can inhibit the activity of glutamate decarboxylase, an enzyme that converts glutamate to γ-aminobutyric acid (GABA). GABA is an important inhibitory neurotransmitter in the central nervous system. A decrease in GABA levels due to the inhibition of glutamate decarboxylase by methylguanidine can lead to an imbalance between excitatory and inhibitory neurotransmitters in the brain, resulting in increased neuronal excitability. This can cause symptoms such as seizures, tremors, and muscle spasms in uremic patients.

Guanidinosuccinic acid can also cause oxidative stress in neurons. It can promote ROS generation in neurons, which can damage the cell membrane, mitochondria, and DNA of neurons. The damage to mitochondria can lead to a decrease in energy production in neurons, affecting their normal function. The damage to DNA can cause mutations and cell death, which can contribute to the development of neurodegenerative diseases in uremic patients. In addition, guanidinosuccinic acid can also affect the function of ion channels in neurons, leading to abnormal electrical activity and cognitive impairment.

Some medium-molecule and protein-bound toxins can also have an impact on the nervous system. For example, B2M, a medium-molecule toxin, can accumulate in the brain in uremic patients. High levels of B2M can result in the formation of amyloid-like deposits in the brain, which can cause neurodegenerative diseases such as dialysis-related amyloidosis. Protein-bound toxins, such as indoxyl sulfate, can also cross the blood–brain barrier and affect neuronal function, although the exact mechanism is still not fully understood. These toxins’ effects on the nervous system can lead to a decline in cognitive function, memory loss, and even uremic encephalopathy in uremic patients, seriously affecting their quality of life.

## 4. Clearance Technologies for Uremic Toxins [5,16]

### 4.1. Traditional Dialysis Methods

#### 4.1.1. Low-Flux Hemodialysis (LFHD)

Low-flux hemodialysis (LFHD) is one of the most commonly used traditional dialysis methods for uremic patients. In LFHD, a semi-permeable membrane is used to separate the patient’s blood from the dialysis fluid. The main mechanisms of toxin removal in LFHD are diffusion and ultrafiltration. During the dialysis process, small-molecule uremic toxins with a molecular weight of ≤500 Da, such as creatinine, urea, and uric acid, pass through the pores of the dialysis membrane via diffusion. The concentration gradient between the blood and the dialysis fluid drives the movement of these small-molecule toxins from the blood to the dialysis fluid, achieving the purpose of clearance. For example, in a study of LFHD treatment for uremic patients, it was found that the clearance rate of creatinine can reach a particular level. However, for medium-molecule toxins with a molecular weight of >500 Da and protein-bound toxins, LFHD has significant limitations [16].

The pore size of the dialysis membrane in LFHD is relatively small, usually around 3–5 nm. This small pore size restricts the passage of medium-molecule toxins. As a result, the clearance of medium-molecule toxins, such as B2M, parathyroid hormone, and interleukin-6, is very limited. For instance, the clearance rate of B2M using LFHD is extremely low, often less than 20%. This is because the molecular size of B2M (11,818 Da) is much larger than the pore size of the LFHD membrane, making it difficult for B2M to pass through the membrane via diffusion.

Protein-bound toxins also pose a challenge to LFHD. These toxins are bound to proteins, forming large-molecular-weight complexes. As LFHD mainly relies on diffusion for toxin clearance, protein-bound toxins cannot easily cross the dialysis membrane. For example, indoxyl sulfate, a typical protein-bound toxin with a high protein-binding rate (more than 80%), has a very low clearance rate in LFHD. The low clearance of protein-bound toxins by LFHD can lead to their continuous accumulation in the body, causing long-term damage to various organs and systems in uremic patients.

#### 4.1.2. High-Flux Hemodialysis (HFHD)

High-flux hemodialysis (HFHD) represents an improvement over LFHD in the clearance of uremic toxins. The key difference between HFHD and LFHD lies in the dialysis membrane. HFHD uses a dialysis membrane with a larger pore size, usually around 6–8 nm, and it has a higher ultrafiltration coefficient. This allows for a higher flux of solutes, providing HFHD with a better ability to clear medium-molecule toxins than LFHD.

The increased pore size in HFHD facilitates the removal of medium-molecule toxins. For example, B2M, which is difficult to clear with LFHD, can be removed to a certain extent using HFHD. The higher clearance rate of HFHD for B2M is due to the larger pore size of the membrane, which allows B2M to pass through the membrane more easily via diffusion and convection.

HFHD also shows advantages in the clearance of some protein-bound toxins. Although protein-bound toxins are still difficult to completely clear with HFHD, the larger pore size and the presence of some adsorption properties in HFHD membranes can increase the clearance efficiency to a certain extent.

However, HFHD also has limitations. For larger-sized medium-molecule toxins or those with a very high protein-binding rate, its clearance efficiency is still not satisfactory. For example, for some cytokines with a molecular weight close to or above 20,000 Da, such as interleukin-18 and tumor necrosis factor-α, the clearance rate of HFHD is relatively low. In addition, HFHD may also cause some side effects. Due to the larger pore size of the membrane, there is a risk of back-filtration of the dialysis fluid, which may introduce contaminants or bacteria into the patient’s blood, increasing the risk of infection.

Although HFHD has improved performance over LFHD in the clearance of medium-molecule toxins, there is still room for improvement, especially for larger-sized and protein-bound toxins. This indicates that, for a more comprehensive and effective clearance of uremic toxins, other advanced blood purification technologies may be needed in combination with HFHD.

### 4.2. Advanced Clearance Technologies

#### 4.2.1. High-Cutoff Membrane Dialysis

High-cutoff dialysis is a relatively advanced blood purification technology. It uses a dialysis membrane with a larger cutoff molecular weight, usually around 50–100 kDa [17]. This technology shows significant advantages in the clearance of large-molecule toxins. For example, it can effectively remove large middle-molecule toxins with a molecular weight of >25–58 kDa, such as pentraxin-3, sTNFR1, AGEs, FGF 23, lambda-FLC, CX3CL1, CXCL12, IL-2, and YKL-400. In a clinical study, it was found that high-cutoff dialysis could significantly reduce the levels of these large-molecule toxins in uremic patients’ blood, which was beneficial for improving their condition.

However, high-cutoff dialysis also has some problems. One of the main issues is the non-selective clearance of albumin. Albumin is an important protein in the blood, playing a crucial role in maintaining colloid osmotic pressure and transporting various substances. Due to the large pore size of the high-cutoff dialysis membrane, albumin can also be non-selectively cleared during the dialysis process. In some cases, the loss of albumin can reach a significant level, which may lead to hypoalbuminemia in patients. Hypoalbuminemia can cause a series of problems, such as edema, reduced immune function, and an increased susceptibility to infections. Therefore, when using high-cutoff dialysis, it is necessary to closely monitor the albumin level in the patient’s blood and take appropriate measures, such as administering albumin supplements, to address the potential negative impacts of albumin loss.

#### 4.2.2. Medium-Cutoff Membrane Dialysis

Medium-cutoff membrane dialysis uses a membrane with a cutoff molecular weight between that of low-flux and high-cutoff membranes, usually around 10–50 kDa. This technology has unique characteristics. It can effectively clear small middle-molecule toxins with a molecular weight of 0.5–15 kDa, such as B2M and IL-8. In a clinical trial comparing medium-cutoff membrane dialysis with traditional low-flux hemodialysis, it was found that medium-cutoff membrane dialysis could achieve a higher clearance rate for B2M, which is beneficial for preventing dialysis-related amyloidosis caused by the accumulation of B2M.

Medium-cutoff membrane dialysis also has good clearance effects on some medium middle-molecule toxins with a molecular weight of >15–25 kDa, such as TNF, IL-18, IL-10, IL-6, kappa-FLC, myoglobin, STNFR2, FGF-2, and prolactin. By effectively removing these toxins, medium-cutoff membrane dialysis can help reduce the inflammatory response and improve the overall condition of uremic patients.

Medium-cutoff membrane dialysis has great potential for clinical application in the future. As the understanding of uremic toxins and the development of membrane materials continue to progress, the performance of medium-cutoff membranes is expected to be further improved. For example, new membrane materials with better biocompatibility, higher permeability, and more selective adsorption properties may be developed, which can further enhance the clearance efficiency of medium-cutoff membrane dialysis for specific toxins and reduce the occurrence of side effects. This technology may become an important part of the comprehensive treatment of uremia, providing more effective treatment options for uremic patients.

#### 4.2.3. Hemoadsorption Technology

Hemoadsorption technology has unique advantages in the clearance of protein-bound toxins [18]. This technology uses adsorbents with a specific affinity for protein-bound toxins. For example, some adsorbents (such as HA130 resin adsorbent from Jafron Biomedical) are designed to specifically target indoxyl sulfate and p-cresol sulfate. These adsorbents can selectively bind to protein-bound toxins, effectively removing them from the patient’s blood. In some clinical studies, when hemoadsorption technology was used in combination with dialysis for uremic patients, it was found that the indoxyl sulfate and p-cresol sulfate levels in the patients’ blood significantly reduced [19,20,21].

The combination of HA and HD can achieve better treatment effects. HA technology can also be combined with other advanced blood purification technologies, such as high-cutoff dialysis, medium-cutoff membrane dialysis, and hemodiafiltration (HDF). For example, clinical studies have demonstrated that HA130 resin-based HA combined with HD significantly enhances the removal of PBUTs. In a randomized controlled study, 136 patients undergoing maintenance hemodialysis (MHD) were divided into four groups: LFHD, HFHD, HDF, and HA+LFHD. The results showed that, for protein-bound toxins with a low affinity to human serum albumin, all four methods had clearance effects similar to those for small-molecule toxins. For toxins with a medium protein-binding affinity (e.g., IS and PCS), HDF (41–47%) and HA+LFHD (45–49%) demonstrated superior removal capabilities to HFHD and LFHD. When it came to toxins with a high protein-binding affinity (e.g., 3-carboxy-4-methyl-5-propyl-2-furanpropanoic acid, CMPF), HA+LFHD showed the best clearance performance, followed by HDF, while LFHD and HFHD were barely effective [22].

The combination of HA (e.g., HA130) and dialysis can achieve synergistic effects. For instance, in a randomized controlled study, 40 MHD patients with uremic pruritus were randomly divided into two groups: an HA130+HD group and an HA130+HDF group. Both groups showed significant decreases in BUN, serum creatinine, phosphates, iPTH, and β2-microglobulin after treatment, with more significant decreases in the HA130+HDF group. Additionally, the pruritus remission rates were higher in the HA130+HDF group (100%) than in the HA130+HD group (75%), indicating that HA+HDF may be a more effective treatment for uremic pruritus [23].

In another randomized controlled trial, 40 MHD patients were divided into three groups: HD only (14 patients), biweekly HA+HD (14 patients), and weekly HA+HD (12 patients) groups. The results showed that the biweekly HA+HD group demonstrated the largest decrease in blood urea nitrogen (BUN) levels and the most significant improvement in pruritus scores. Only the biweekly HA+HD group showed significant reductions in CRP levels, appetite scores, and pruritus scores after therapy. Both the biweekly HA+HD and weekly HA+HD groups exhibited significant improvements in sleep quality scores after therapy. Overall, the study concluded that combining HD with biweekly HA therapy can significantly reduce inflammatory markers and improve symptoms in ESRD patients [24].

This multi-technology combination can achieve a more comprehensive and efficient clearance of different types of uremic toxins, providing a more personalized and effective treatment plan for uremic patients. The development of adsorption technology has opened up new avenues for the treatment of uremia, and, with the continuous improvement of technology, it is expected to play an increasingly important role in the clinical treatment of uremic patients.

Table 4 and Table 5 present a comparison of different dialysis modalities in terms of their clearance rates for several common uremic toxins.

## 5. Integration of Toxin Classification, Toxicity and Clearance Technologies

### 5.1. The Correlation Between Classification and Clearance Strategies [7]

Understanding the correlation between the classification of uremic toxins and clearance strategies is crucial for the effective treatment of uremic patients [25]. Different types of toxins require specific clearance methods due to their unique physicochemical properties and biological activities.

Small-molecule toxins, which have a small molecular size and high water solubility, are relatively easy to clear using traditional dialysis methods. As previously mentioned, creatinine, urea, and uric acid can be effectively removed through diffusion using low-flux hemodialysis (LFHD) and high-flux hemodialysis (HFHD). LFHD, which uses a membrane with a small pore size, can still achieve a relatively high clearance rate for these small-molecule toxins because their molecular size allows them to pass through the membrane pores.

However, medium-molecule toxins pose more challenges. Their larger molecular size makes them difficult to clear using LFHD. However, for some larger-sized medium-molecule toxins, HFHD is still insufficient. High-cutoff dialysis and medium-cutoff membrane dialysis technologies are more suitable for medium-molecule toxins. High-cutoff dialysis, which has a membrane cutoff molecular weight of around 50–100 kDa, can effectively remove large middle-molecule toxins with a molecular weight of >25–58 kDa. Medium-cutoff membrane dialysis, which has a cutoff molecular weight of around 10–50 kDa, is very effective in clearing small middle-molecule toxins with a molecular weight of 0.5–15 kDa, such as B2M and IL-8. These advanced dialysis technologies can better meet the clearance requirements of middle-molecule toxins due to their specific membrane characteristics.

Protein-bound toxins are the most difficult to clear. Their high protein-binding rate and the formation of large-molecular-weight complexes after binding to proteins make traditional dialysis methods almost ineffective. Adsorption technology is a key solution for protein-bound toxins. Adsorbents with a specific affinity for protein-bound toxins can selectively bind to them and effectively remove them from the blood.

In summary, understanding the correlation between toxin classification and clearance strategies is essential for optimizing the treatment of uremic patients. By selecting the appropriate clearance method according to the characteristics of different toxins, it is possible to achieve more efficient toxin removal, reduce the toxicity of uremic toxins to the body, and improve the treatment effect and quality of life of uremic patients.

### 5.2. The Guiding Role of Toxicity Assessment in Treatment [5]

The results of toxicity assessments, which are essential for improving the safety and effectiveness of treatment for uremic patients, play a crucial guiding role in the clinical development of personalized treatment plans.

For toxins with a high C_U_/C_N_ ratio, such as guanidinosuccinic acid and indoxyl sulfate, their high-level presence in uremic patients indicates a significant contribution to toxicity. In the case of patients with high levels of indoxyl sulfate, which are closely related to cardiovascular damage, personalized treatment plans need to focus on effectively reducing its concentration. As traditional dialysis methods have limited clearance capacities for indoxyl sulfate, they can be combined with adsorption-based techniques. For example, specific adsorbents with a high affinity for indoxyl sulfate can be used in combination with dialysis. This can not only enhance the clearance of indoxyl sulfate but also reduce the risk of cardiovascular complications, improving the safety and effectiveness of treatment. In a clinical study, when adsorption-enhanced dialysis was used for patients with high indoxyl sulfate levels, their cardiovascular function improved significantly, and the incidence of related complications decreased.

Toxins with a high C_MAX_/C_U_ ratio, such as γ-guanidinobutyric acid, pose greater risks in specific patients due to their uneven distribution. For these patients, the close monitoring of toxin levels is necessary. In addition, personalized treatment strategies can be adjusted according to the patient’s genetic background, gut microbiota status, and other factors. If a patient has a genetic predisposition that affects the metabolism of γ-guanidinobutyric acid, relevant drugs can be used to regulate its metabolism while using appropriate blood purification methods. For example, if the patient’s gut microbiota is found to be abnormal and this is suspected to be related to the high-level accumulation of γ-guanidinobutyric acid, probiotics can be used to adjust the gut microbiota, combined with advanced blood purification technologies such as high-cutoff dialysis or medium-cutoff membrane dialysis, depending on the molecular weight characteristics of the toxin. This can help to more precisely control the toxin level, reduce the potential harm to the patient, and improve the treatment effect.

In patients with symptoms related to uremic toxins, such as pruritus or restless leg syndrome, toxicity assessment can help identify the specific toxins responsible. If the toxicity assessment shows that certain protein-bound toxins are the main cause of pruritus, then targeted treatment strategies can be developed. In addition to using adsorption technology to remove these protein-bound toxins, drugs that can relieve itching symptoms can also be used in combination. This comprehensive treatment approach can not only reduce the toxicity of toxins but also directly alleviate the patient’s symptoms, improving their quality of life. Overall, toxicity assessment provides a scientific basis for personalized treatment, enabling doctors to make more accurate treatment decisions, which is of great significance for improving the treatment outcome of uremic patients.

## 6. Research Gaps and Future Perspectives

The 20 years of research progress from 2003 to 2023 has resulted in clearer and more scientific molecular classifications—transitioning from simple uremic toxin classifications to new classification dimensions (focusing on the multidimensional effects of toxins, such as the impact on target organs, patient symptoms and clinical outcomes, and toxin clearance techniques and efficiency).

The integration of hemoadsorption with conventional hemodialysis represents a transformative approach to overcoming the persistent challenges in clearing protein-bound uremic toxins (PBUTs), such as indoxyl sulfate and p-cresol sulfate, which exhibit a high protein-binding affinity and resistance to standard dialysis modalities. The current difficulties in clearance and unmet clinical needs, including adequate PBUT clearance and the improvement or prevention of associated cardiovascular, inflammatory, and metabolic complications, underscore the limitations of isolated HD strategies.

HA+HD synergistically addresses these gaps by leveraging hemoadsorption technologies—such as porous resin or polymer-based adsorbents—to target PBUTs through hydrophobic and electrostatic interactions, while HD maintains the efficient removal of small water-soluble toxins. This dual-modality approach enhances the efficiency of toxin clearance, particularly for high-risk solutes with molecular weights of >15 kDa or strong protein-binding properties (>80%), thereby mitigating their cumulative impact on target organs. Emerging innovations further emphasize the integration of the real-time monitoring of biomarkers (e.g., urea, creatinine, B2M, iPTH, oxidative stress markers) to dynamically optimize HA+HD protocols, enabling personalized blood purification tailored to individual toxin profiles and clinical outcomes. By bridging the evolved molecular classification with advanced clearance strategies, the combination of HA+HD exemplifies a paradigm shift toward precision medicine in nephrology, offering a scalable solution to reduce the long-term morbidity and improve the quality of life of end-stage kidney disease patients.

### 6.1. Existing Problems in Current Research

The current research into uremic toxins has several notable limitations. Firstly, the detection methods for uremic toxins are still far from perfect [26]. Existing detection techniques mainly rely on a limited number of biomarkers, such as urea, creatinine, and phosphate. These biomarkers are not sufficient to comprehensively reflect the complex situation of uremic toxins in patients. For example, they cannot accurately reflect the levels of many medium-molecule and protein-bound toxins. The detection of medium-molecule toxins such as B2M and cytokines often requires more sensitive and specific methods. Protein-bound toxins, due to their complex binding states and low concentrations in the free form, are even more difficult to accurately detect. The lack of accurate detection methods makes it challenging to precisely evaluate the toxicity of uremic toxins and monitor the treatment effect, which may lead to suboptimal treatment decisions.

Secondly, there are bottlenecks in the development of clearance technology for uremic toxins. Although there are various advanced clearance technologies, they still face challenges. For example, although high-cutoff dialysis can effectively remove large-molecule toxins, its non-selective clearance of albumin is a major drawback. Albumin loss can cause a series of problems such as hypoalbuminemia, which may lead to edema, reduced immune function, and an increased susceptibility to infections in patients. Medium-cutoff membrane dialysis also has limitations. Although it can clear some medium-molecule toxins, its clearance efficiency for larger-sized medium-molecule toxins or those with complex structures may not be satisfactory. Adsorption technology, despite its advantages in clearing protein-bound toxins, still has room for improvement in terms of the selectivity and capacity of adsorbents. Some adsorbents may not be able to fully and selectively remove all types of protein-bound toxins, and their saturation capacity may limit the continuous removal of toxins during long-term treatment.

Finally, the current research into personalized treatment regimens for uremic patients is insufficient. The classification of uremic toxins and the development of clearance strategies have not been fully integrated with the individual characteristics of patients. Patients may have different genetic backgrounds, gut microbiota compositions, and comorbidities, which can affect the production, metabolism, and toxicity of uremic toxins. However, most of the current treatment strategies are relatively uniform and do not fully consider these individual differences. For example, in patients with different genetic polymorphisms related to toxin metabolism, the same treatment method may have different effects. In addition, the impact of gut microbiota on the production of protein-bound toxins from gut microbial metabolism is also not well considered in personalized treatment. Without personalized treatment regimens, it is difficult to achieve the best treatment effect for each uremic patient, and it may also lead to unnecessary treatment costs and side effects.

### 6.2. Prospects for Future Research

In the future, research into uremic toxins is expected to focus on several key aspects [27]. Firstly, the development of novel toxin detection methods is crucial. Scientists may explore the use of advanced analytical techniques such as mass spectrometry-based multi-omics technology. This technology can allow for the simultaneous analysis of the metabolome, proteome, and lipidome in the blood of uremic patients, enabling the detection of a wide range of uremic toxins, including small-molecule, medium-molecule, and protein-bound toxins. For example, liquid chromatography–tandem mass spectrometry (LC-MS/MS) can be used to accurately identify and quantify various toxins in complex biological samples. By analyzing the mass spectra of samples, researchers can determine the chemical structures and concentrations of toxins, providing more comprehensive information for toxicity assessment and treatment monitoring [28].

Secondly, the optimization of dialysis membranes and adsorption materials is an important research direction. For dialysis membranes, new materials with better biocompatibility, higher permeability, and more selective adsorption properties may be developed. For example, nanofiber-based dialysis membranes can be designed to have precise pore sizes and surface chemistries. These membranes could selectively remove specific toxins while minimizing the loss of beneficial substances such as albumin. In terms of adsorption materials, the development of novel adsorbents with a higher affinity and selectivity for protein-bound toxins is expected.

Finally, the application of artificial intelligence (AI) and machine learning in the treatment of uremia shows great potential. AI can be used to analyze large-scale patient data, including toxin profiles, clinical symptoms, and treatment responses. By using machine learning algorithms, AI can predict the toxicity of different toxins in individual patients and then recommend personalized treatment strategies. For example, a neural network-based model can be trained with a large amount of patient data to predict the risk of cardiovascular events in uremic patients based on their toxin levels. According to the prediction results, doctors can adjust the treatment plan in a timely manner, for example, by increasing the frequency of dialysis or using specific adsorption therapies. AI can also be used to optimize the design of dialysis membranes and adsorption materials. By simulating the interaction between toxins and materials, AI can guide the development of more efficient and selective blood purification materials, ultimately achieving precise treatment for uremic patients.

## 7. Conclusions

### 7.1. Summary of Key Points

In summary, research into uremic toxins has advanced significantly. The toxin classification evolved from the 2003 EUTox system, which focuses on molecular weight and protein binding, to more comprehensive systems introduced in 2021 and 2023. The 2021 consensus classification considers toxin clearance technologies, organ impacts, and clinical outcomes, while the 2023 classification emphasizes patient outcomes, novel toxin identification, and advanced technologies such as dialyzer membranes and AI-based prediction models. Toxicity assessments using indicators such as C_U_/C_N_ and C_MAX_/C_U_ ratios have provided insights into the toxic effects of different toxins. Traditional dialysis methods such as LFHD and HFHD have limitations, while advanced clearance technologies such as high-cutoff dialysis, medium-cutoff dialysis, and hemoadsorption technology can address these limitations. However, it is worth noting that high-cutoff dialysis non-selectively clears albumin, imposing a burden on patients instead; conversely, hemoadsorption is a relatively safe and effective therapy in this case, and the combined HA+HD modality is expected to be the optimal combination for targeted clearance.

It is recognized that different toxins have different clearance characteristics and that the choice of clearance technology should be based on their specific properties. Therefore, understanding the correlation between toxin classification and clearance strategies is crucial for achieving personalized therapy and may eventually lead to improvements in patients’ quality of life and survival rates.

### 7.2. Significance for Clinical Practice and Future Research

This review has important implications for clinical practice. It provides a comprehensive understanding of uremic toxins, aiding clinicians in assessing patient conditions and selecting appropriate treatment methods. In terms of future research, this review highlights the need for novel toxin detection methods, the optimization of dialysis membranes and adsorption materials, and the application of AI and machine learning to improve treatment outcomes. The concept of precise blood purification treatment is expected to become more personalized, efficient, and safe through continuous research and innovation.

## Figures and Tables

**Table 1:** The 2003 EUTox classification and clearance modalities (Vanholder, Raymond, EUTox Work Group et al., 2003) [5].

Category	Examples	Clearance Efficiency	Clinical Impact
Small-molecule toxins (<500 Da, 45 types)	Urea, creatinine, uric acid	High (LFHD)	Metabolic derangements
Middle-molecule toxins (≥500 Da, 22 types)	β_2_-MG, IL-6, PTH	Moderate (HFHD/HDF)	Bone disease, inflammation
Protein-bound toxins (25 types)	IS, PCS, AGEs	Low (conventional dialysis)	CVD, endothelial dysfunction

**Table 2:** Differences between 2003 EUTox and 2021 consensus classifications.

Items	2003 EUTox	2021 Consensus
**Chemical identification and quantitative analysis**	It is stated that toxins must be chemically identified and accurately quantified in biological fluids. However, the terms “biological fluids” and “chemical identification” are overly broad and nonspecific.	It is stated that toxins need to be identifiable and quantifiable in plasma, serum, or blood, with the analysis objects specified.
**Measurement of total toxin levels and plasma concentrations**	It is stated that the total amount and plasma concentration of toxins in uremic patients should be higher than those in non-uremic patients. However, it is not clarified whether the total amount of toxins can be accurately measured.	It is recommended to compare the levels in plasma, serum, or blood with those of individuals with normal renal function, and it is pointed out that toxin levels in patients with chronic kidney disease (CKD) should be higher than those in individuals with normal renal function.
**Relationship between toxin concentrations and clinical symptoms**	It is believed that high concentrations of toxins are associated with specific uremic symptoms and/or functional impairments, and improvements should occur when concentrations decrease.	It is proposed that a reduction in concentration does not necessarily translate into clinical improvement. It should be determined whether biological and clinical changes are related to changes in toxin concentrations.
**Standards for toxin concentrations**	It is also mentioned that these associations should align with the concentrations of toxins in the body fluids or tissues of uremic patients.	It is emphasized that the biologically active concentrations in research should be consistent with the plasma, serum, or blood concentrations of CKD patients, avoiding the use of nonspecific terms (e.g., “uremia”).

**Table 3:** High-impact toxins by systemic effects.

Toxin Class	Key Toxins	Primary Pathophysiology	Clearance Strategy
Protein-bound	IS, PCS, AGEs	Oxidative stress, endothelial dysfunction	Adsorption, high-cutoff dialysis
Middle-molecule	β2-MG, FGF-23, TNF-α	Chronic inflammation, bone mineral disorder	HFHD, middle-cutoff dialysis
Small-molecule	ADMA, trimethylamine N-oxide (TMAO)	Vascular calcification, platelet dysfunction	LFHD + pharmacological intervention

**Table 4:** Comparison between different dialysis modalities in terms of clearance rates.

Molecular Weight	Exogenous Molecules		Endogenous Water-Soluble Molecules			
	Gut-Derived, Protein-Bound Molecules < 80%	Gut-Derived, Protein-Bound Molecules ≥ 80%	Small Molecules (<0.5 kDa)	Small–Middle Molecules (0.5–15 kDa)	Medium–Middle Molecules (15–25 kDa)	Large–Middle Molecules (25–58 kDa) and Large Molecules (58–170 kDa)
Uremic toxins	ADMA, SDMA, uric acid, carbamylated compounds, urea, TMAO	Hcy, IS, pCS, CML, kynurenines	Uric acid, urea	β2-microglobulin, IL-8	TNF, IL-18, IL-10, IL-6, kappa-FLC, myoglobin, sTNFR2, FGF-2, prolactin, complement factor D	AGEs, FGF-23, lambda-FLC, albumin
Low-flux HD	YES	**NO**	YES	**NO**	**NO**	**NO**
High-flux HD	YES	**NO**	YES	YES	**NO**	**NO**
Online HDF HDx	YES	YES	YES	YES	YES	**NO**
**Hemoadsorption (HA)**	**YES**	**YES**	**YES**	**YES**	**YES**	**YES**

**Table 5:** Clearance efficiency by modality.

Toxin Category	HD	HA	HA+HD
**Small Molecules**	+++	+	+++
**Middle Molecules**	++	++	+++
**Protein-bound Toxins**	+	+++	+++

+++ very high; ++ high; + moderate.
